# Wavefront manipulation based on transmissive acoustic metasurface with membrane-type hybrid structure

**DOI:** 10.1038/s41598-018-32547-3

**Published:** 2018-09-21

**Authors:** Jun Lan, Xiaowei Zhang, Xiaozhou Liu, Yifeng Li

**Affiliations:** 1 Key Laboratory of Modern Acoustics, Institute of Acoustics and School of Physics, Collaborative Innovation Center of Advanced Microstructures, Nanjing University, Nanjing, 210093 P. R. China; 2 College of Computer Science and Technology, Nanjing Tech University, Nanjing, 211800 P. R. China

## Abstract

We designed and demonstrated a gradient acoustic metasurface to manipulate the transmissive wavefront. The gradient metasurface is composed of eight elements based on membrane-type hybrid structures, whose thickness and width are about 1/5 and 1/20 of the incident wavelength, respectively. Here, we employ acoustic theory to analyze the transmission spectrum and phase gradient of the metasurface, the properties of high transmission efficiency and discrete phase shifts over the full $2\pi$ range can be achieved simultaneously. By appropriate selection of the phase profile along the transverse coordinate of the metasurface or the angle of incident wave, the transmissive wavefront manipulations based on metasurface can be obtained as expected from the generalized Snell’s law, such as anomalous refraction, acoustic cloak based on flat focusing, acoustic self-bending beam, conversion of propagating wave to surface wave and negative refraction. Our gradient metasurface may have potential application in low-loss acoustic devices.

## Introduction

Recently, the acoustic metasurfaces as a family of wavefront-shaping devices with planar profile have attracted tremendous interest. By introducing the discrete phase variations from 0 to $2\pi$ across the surface, the acoustic metasurface of subwavelength thickness is capable of many forms of wavefront manipulations. In general, the generalized Snell’s law is widely utilized to provide accurate phase response of the metasurface and interpret the unconventional wavefront phenomena. By using an acoustic metasurface with transversal gradient phase or velocity profile, various unique phenomena or properties have been revealed, such as anomalous refraction/reflection^1,2^, acoustic bending^3^, subwavelength flat focusing^4,5^, asymmetric propagation^6,7^ and propagating wave converting into surface wave^8^.

For the purpose of flexibly tailoring the propagation of the transmitted acoustic wave, the elements of the acoustic metasurface should have two critical properties^9–11^. First, full control of the phase of acoustical field is required, i.e., shaping the phase over a complete $2\pi$ range. However, due to the limitation of the acoustic properties in the existing acoustic material, there still remains challenge to realize the phase changes covering the full $2\pi$ range^12^. Highly efficient transmission is another critical property, which can be fulfilled by the locally resonant or geometry based non-locally resonant elements. For the former, examples of using membrane based elements^13–17^ or Helmholtz resonator (HR) based elements^6,9,10,18–20^ have been proposed. It is noteworthy that good impedance matching can be obtained around the resonant frequency. Non-locally resonant elements can be designed by the tapered labyrinthine structure, the gradually varying cross-sectional area of the tapered structure has diminished the impedance mismatch caused by the sudden change of cross-sectional area^4,21–23^. Despite most of previous metasurfaces designed with wavefront manipulations, it is still difficult to take the two properties into account simultaneously. However, in this paper, the connection of four decorated membrane resonators provides an effective acoustic reactance to shift the phase of the incident acoustic wave over the whole $2\pi$ range and realize the highly efficient transmission.

The membrane-type acoustic metasurfaces consisting of decorated membrane resonators of various forms have been widely studied^24–27^. However, almost all previous studies were focused on employing this structure to achieve robust impedance matching and perfect absorption. On the basis of this feature, in this paper, the decorated membrane resonator is introduced to design a transmissive acoustic metasurface. We use eight elements to construct the metasurface, and each element is designed by the membrane-type hybrid structure composed of four decorated membrane resonators in periodical distribution and a straight pipe with tunable width. With the acoustic transmission line method (ATLM) and impedance theory, we can derive that the decorated membrane resonators possess the abilities of shaping the phase over the full $2\pi$ range and overcoming the impedance mismatching to enhance sound transmission. Furthermore, the distinct wavefront manipulations such as anomalous refraction, acoustic cloak based on flat focusing, acoustic self-bending beam, conversion of propagating wave to surface wave and negative refraction are demonstrated based on the generalized Snell’s law. These five wavefront manipulations are enabled by free manipulation of the transversal phase profile along the metasurface or the angle of incident wave.

## Results

### Analytical model of the acoustic metasurface element

**Figure 1:**
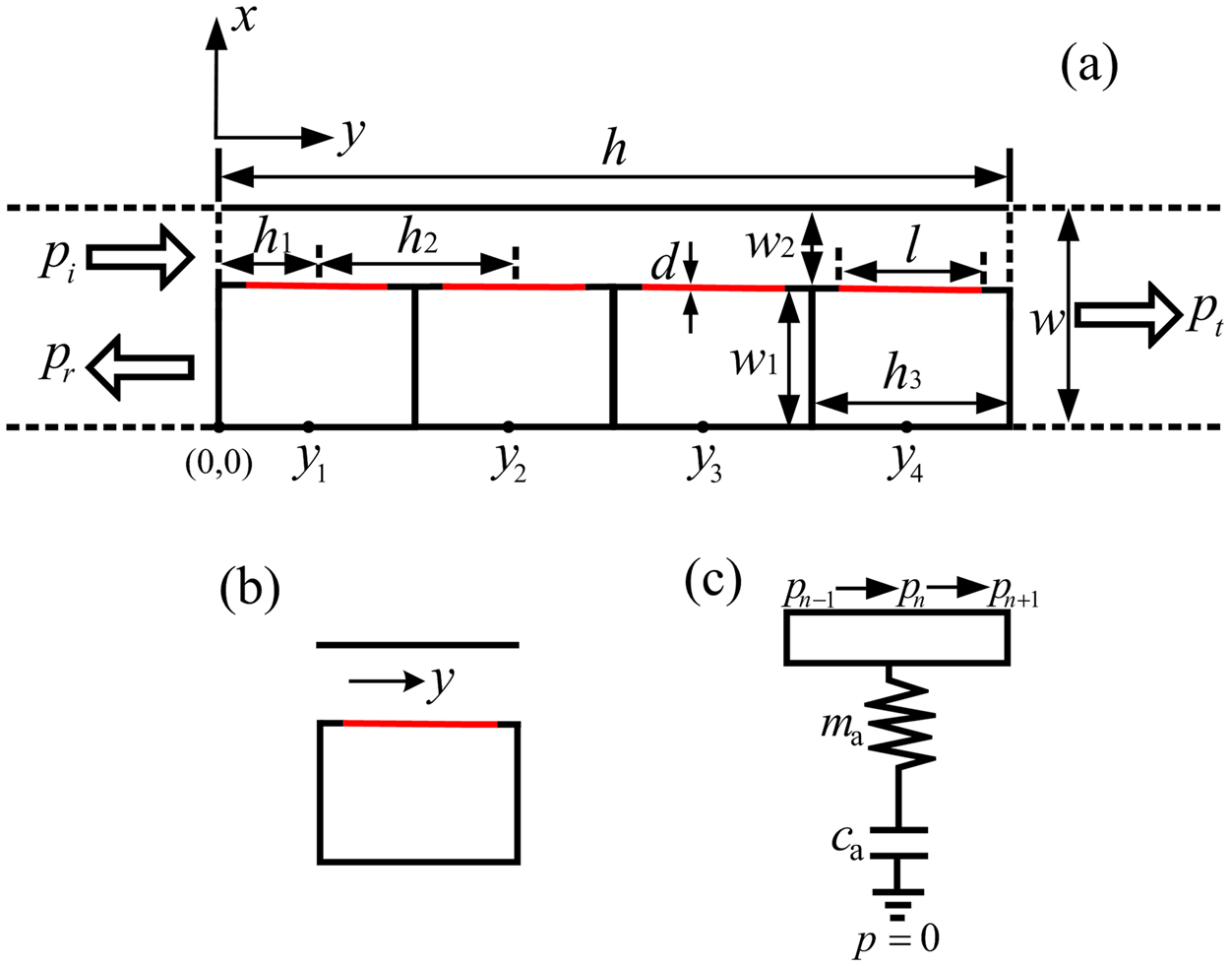
Schematic diagram illustration of the acoustic metasurface element. (**a**) Schematic illustration of an individual element of the metasurface made of four decorated membrane resonators and a straight pipe. Red solid lines refer to membranes. (**b**) The cross-sectional picture of a single cell. (**c**) The equivalent acoustic transmission line circuit of a cell.

We firstly demonstrate the construction of the acoustic gradient metasurface. Figure 1(a) is the schematic diagram of an individual element of the acoustic metasurface in the *xy*-plane. The element is designed by the membrane-type hybrid structure consisting of four decorated membrane resonators arranged in *y* direction with periodic constant *h*_2_ (*h*_2_ = 2*h*_1_) and a straight pipe with tunable width *w*_2_. The decorated membrane resonator is composed of a rigid back cavity filled with air and a membrane with width *l* = 9 mm and thickness *d* = 0.1 mm. The membrane’s edges are ideally fixed on the ends of side walls to seal the cavity. The cavity with a fixed height *h*_3_ = 10 mm and a tunable width *w*_1_ to span the phase over a $2\pi$ range. The external dimensions of the element are thickness *h* = 40 mm and width *w* = 10 mm. The mass density (${\rho }_{{\rm{m}}}$), Young’s modulus (*E*), and Poisson’s ratio ($\nu$) of the membrane are 1420 kg/m^3^, 1 GPa, and 0.34, respectively. The background medium is air, whose density ${\rho }_{0}$ is 1.21 kg/m^3^ and acoustic velocity ${c}_{0}$ is 343 m/s.

In order to simplify the analysis, the acoustic metasurface can be described with the equivalent inductance and capacitance resonance^28–30^. Each element of the metasurface is divided into four identical cells. Figure 1(b) shows the cross-section of a single cell, which is composed of a straight pipe section and a decorated membrane resonator. *y* indicates the direction of the sound wave propagation. First, we convert the cell into its equivalent acoustic circuit representation, and the cell could be separated into two parts, i.e., a pipe and a *L*-*C* circle, as shown in Fig. 1(c). The acoustic model of a decorated membrane resonator can be described by an acoustic impedance *Z*_a_, which is consisting of two serial acoustic impedances of membrane *Z*_am_ and rigid back cavity *Z*_ac_ in the acoustic circuit, *Z*_a_ = *Z*_am_ + *Z*_ac_. Generally, the acoustic impedance of the 2-dimentional (2-D) membrane can be defined as ${Z}_{{\rm{am}}}={\iint }_{S}{\rm{\Delta }}pdS/(j\omega \bar{\xi }{S}^{2})$, where ${\rm{\Delta }}p$ is the pressure difference across the membrane, $\bar{\xi }$ is the average transverse displacement of the membrane, and *S* is the cross-sectional area of the membrane^28^. It should be noted that this expression of ${Z}_{{\rm{am}}}$ is only valid for 2-D circular membranes^16,17,31^. In our 1-dimentional (1-D) membrane sample, the cross-sectional area *S* of the membrane is the width of the membrane *l*. Therefore, the acoustic impedance of the membrane is ${Z}_{{\rm{am}}}={\int }_{l}{\rm{\Delta }}pdl/(j\omega \bar{\xi }{l}^{2})$, and the analytical solution for ${Z}_{{\rm{am}}}$ is provided in Supplementary Information. The acoustic impedance of the membrane can be described by a resonant circuit comprised of an acoustic mass ${m}_{{\rm{am}}}$ and an acoustic capacitance ${c}_{{\rm{am}}}$. The air in the rigid back cavity can be regarded as a spring in response to membrane oscillation. Thus, the acoustic impedance of the back cavity acts as a capacity with acoustic capacitance ${c}_{{\rm{ac}}}={w}_{1}{h}_{3}/{\rho }_{0}{{c}_{0}}^{2}$. In this *L*-*C* circle, the membrane contributes to the acoustic mass part of the total acoustic mass. Therefore, the parameters of the *L*-*C* circle in Fig. 1(c) can be given by the effective acoustic mass ${m}_{{\rm{a}}}={m}_{{\rm{am}}}$ and acoustic capacitance ${c}_{{\rm{a}}}={c}_{{\rm{am}}}{c}_{{\rm{ac}}}/({c}_{{\rm{am}}}+{c}_{{\rm{ac}}})$. The expression for the total acoustic impedance of the decorated membrane resonator is ${Z}_{{\rm{a}}}=j(\omega {m}_{{\rm{a}}}-1/(\omega {c}_{{\rm{a}}}))$.

**Figure 2:**
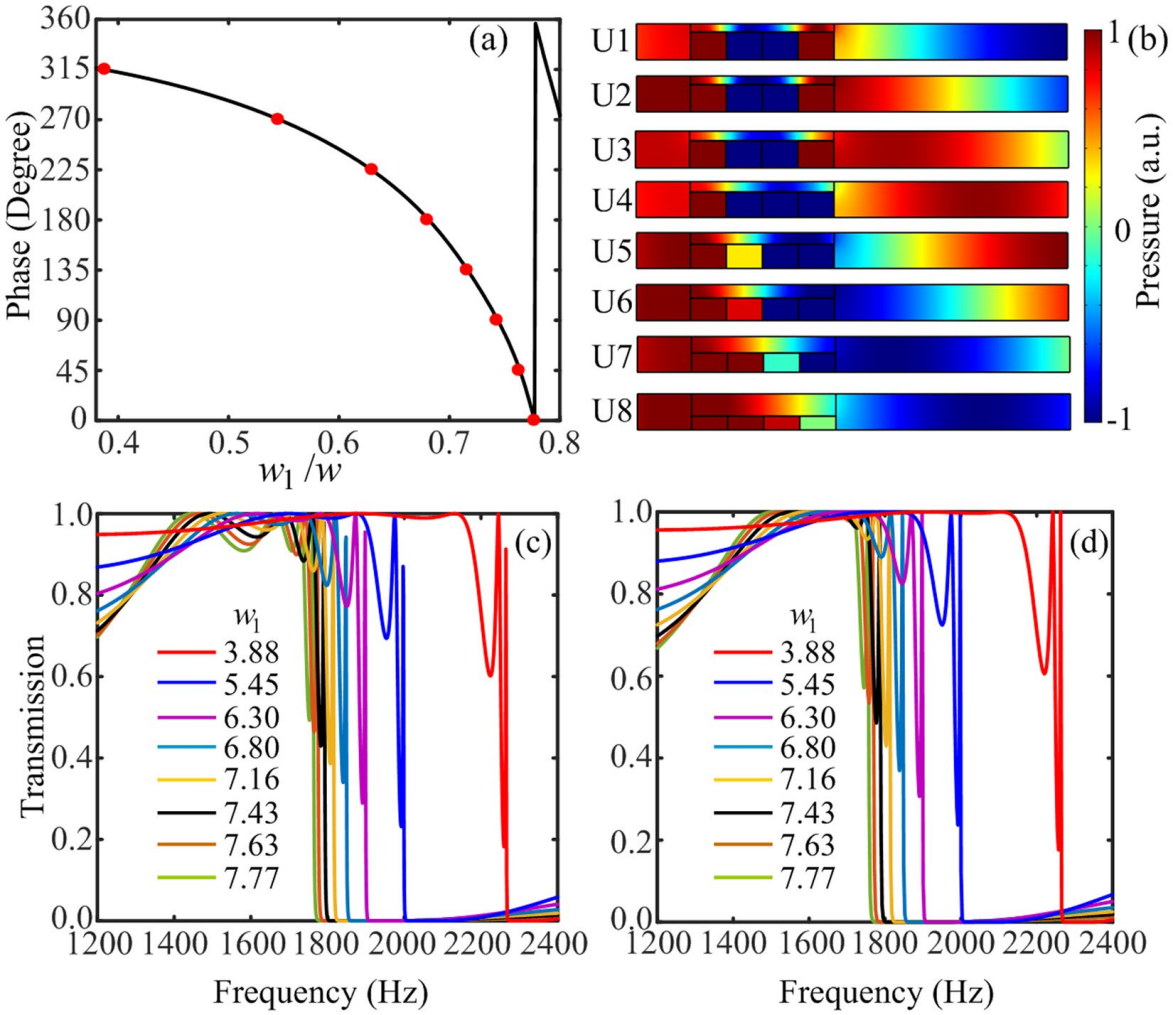
The acoustic wave transmitted properties of the eight elements of acoustic metasurface. (**a**) The phase of the transmitted wave as a function of cavity width ratio *w*_1_/*w* with incident wavelength *λ* = 19.8 cm. (**b**) The pressure field patterns of the transmitted waves for eight elements. (**c,d**) The numerical simulation and theoretical calculation of the sound intensity transmission coefficients as a function of frequency for eight elements.

In order to modify the radiation pattern of the transmitted acoustic wave efficiently, we present how the phase of the acoustic wave is able to modulate by the element consisting of four decorated membrane resonators and a straight pipe. The discussions above have shown that the decorated membrane resonator can be described by a lumped element, which provides an effective acoustic reactance to shift the phase of the incident acoustic wave. However, the phase shift provided by a single decorated membrane resonator is limited within a small range. Therefore, a series connection of four decorated membrane resonators should be employed to achieve a wide range of phase shift^9,10^. Here, we adopt eight elements with fixed transverse dimension *w* = 10 mm, and by tuning the width of the cavity *w*_1_, the width of the pipe *w*_2_ changes as well, so that the effective acoustic reactance provided by the element will change and the phase shift can span over a complete $2\pi$ range. Figure 2(a) illustrates the simulated phase of the transmitted wave as a function of the tunable cavity width ratio *w*_1_/*w* (shown as black curve) at the particular chosen frequency of 1735 Hz, the corresponding acoustic wavelength is *λ* = 19.8 cm. For the sake of the facility, the discrete phase shifts with steps of $\pi /4$ provided by eight elements (shown as red dots) are adopted to replace the continuous phase, and the widths of the cavities *w*_1_ for eight elements are optimized to be 7.77 mm, 7.63 mm, 7.43 mm, 7.16 mm, 6.80 mm, 6.30 mm, 5.45 mm and 3.88 mm, respectively. To further verify the discrete phase shifts covering the full $2\pi$ range with steps of $\pi /4$, the simulated transmitted pressure field patterns for these eight elements are shown in Fig. 2(b). The peak of the pressure field can shift up to a wavelength, which means the discrete phase shifts cover the whole $2\pi$ range. It could be intuitively inferred from Fig. 1(a) that the acoustic impedance of the element could not match with the background medium because of the existence of narrow entrance and exit in the element. However, the impedance matching between the background medium and the element can be achieved for the special frequency band. Figure 2(c) shows the simulated sound intensity transmission coefficients as a function of frequency for eight elements. It is observed that these eight elements are capable of high transmission efficiency for the acoustic wave in the frequency ranging from 1400 Hz to 1750 Hz, since all the transmission coefficients are large than 80% for eight elements. Consequently, we could conclude that the desirable discrete phase shifts and high transmission efficiency can be achieved by these eight elements.

To verify the high transmission efficiency property of the element with acoustic theory, ATLM is introduced to explore the underlying physical mechanism and describe the propagation of acoustic wave in these eight elements^9,32^. Considering a plane wave normally impinges on the element (as shown in Fig. 1(a)) along the +*y* direction. The transmitted wave is formed by the radiation effect of the outlet, and the output impedance of the element is given by ${Z}_{{\rm{al}}}={\rho }_{0}{c}_{0}/w$. According to ATLM, the output impedance of the element can be transferred to the center position of the last decorated membrane resonator using transmission line impedance transfer formula, ${Z^{\prime} }_{y={y}_{4}}={Z}_{0}({Z}_{{\rm{al}}}+j{Z}_{0}\,\tan \,k{h}_{1})/({Z}_{0}+j{Z}_{{\rm{al}}}\,\tan \,k{h}_{1})$, where ${Z^{\prime} }_{y={y}_{4}}$ is the transferred acoustic impedance of the pipe at *y*_4_ = *h*_1_ + 3*h*_2_ and ${Z}_{0}$ is the acoustic impedance of the straight pipe given by ${Z}_{0}={\rho }_{0}{c}_{0}/{w}_{2}$. The transferred acoustic impedance ${Z}_{y={y}_{4}}^{^{\prime} }$ is parallel connected to the fourth decorated membrane resonator, and the parallel acoustic impedance at $y={y}_{4}$ can be expressed as ${Z}_{y={y}_{4}}={Z^{\prime} }_{y={y}_{4}}{Z}_{{\rm{a}}}/({Z^{\prime} }_{y={y}_{4}}+{Z}_{{\rm{a}}})$. Similarly, the parallel acoustic impedance ${Z}_{y={y}_{i+1}}$ (*i* = 3, 2, 1) can be transferred to the center position of the *i*th decorated membrane resonator, etc., the transferred acoustic impedance of the pipe *y*_3_ = *h*_1_ + 2*h*_2_, *y*_2_ = *h*_1_ + *h*_2_ and *y*_1_ = *h*_1_ can be obtained from ${Z^{\prime} }_{y={y}_{i}}={Z}_{0}\times [({Z}_{y={y}_{i+1}}+j{Z}_{0}\,\tan \,k{h}_{2})/({Z}_{0}+j{Z}_{y={y}_{i+1}}\,\tan k{h}_{2})]$, which is parallel connected to a shunt impedance *Z*_a_, and the corresponding acoustic impedance is given by

1 $${Z}_{y={y}_{i}}=\frac{{Z^{\prime} }_{y={y}_{i}}{Z}_{{\rm{a}}}}{{Z^{\prime} }_{y={y}_{i}}+{Z}_{{\rm{a}}}}.$$

Then, the transferred acoustic impedance at the entrance of the element (*y* = 0) can be written as

2 $${Z}_{y=0}={Z}_{0}\frac{{Z}_{y={y}_{1}}+j{Z}_{0}\,\tan \,k{h}_{1}}{{Z}_{0}+j{Z}_{y={y}_{1}}\,\tan \,k{h}_{1}}.$$

Thus, the acoustic pressure reflection coefficient can be obtained to be ${r}_{{\rm{p}}}=({Z}_{y=0}-{Z}_{{\rm{al}}})/({Z}_{y=0}+{Z}_{{\rm{al}}})$, and the sound intensity transmission coefficient is $T=1-{|{r}_{{\rm{p}}}|}^{2}$. Here, we note that ATLM does not need to involve any complex wave equations. Figure 2(d) plots the theoretical sound intensity transmission coefficients as a function of frequency for eight elements. Through comparing it with the simulated result (as shown in Fig. 2(c)), it is found that the overall trend of the transmission coefficients for these eight elements are almost the same. Therefore, the following numerical simulations can be studied based on theoretical analysis. Hence, the designed elements of the metasurface have the capabilities of shaping the phase over the full $2\pi$ range and high transmission efficiency simultaneously.

The acoustic metasurface with elements of a hybrid structure consisting of acoustic HRs and a straight pipe with the $\lambda /2$ length has been explicitly realized. This structure obtained a high transmission and a shifting phase with $2\pi$ span simultaneously^6,9,10,20^. However, owing to the existence of short neck of HR, when the width ratio of the straight pipe to the cavity of the HR is small, the geometrical mismatch between the element and the background medium and low transmission will appear inevitably. Here, we present an acoustic metasurface that consisted of four decorated membrane resonators and a straight pipe with the thickness of $\lambda /5$. It is similar to the HR, the membrane of the decorated membrane resonator corresponds to the short neck of HR and acts as inductor with acoustic mass. In addition, the effect of membrane will diminish geometrical mismatch caused by the short neck and achieve a metasurface with small external dimensions.

### Transmissive wavefront manipulation based on the generalized Snell’s law

As shown above, we have proved that the eight elements support discrete phase shifts over $2\pi$ range with steps of $\pi /4$ and high transmission efficiency. In the following, under the guidance of the generalized Snell’s law, we demonstrate that the acoustic metasurface has the capability of manipulating transmissive wavefront flexibly. The generalized Snell’s law introduces an abrupt phase variation to describe the phase gradient along the metasurface, as follows

3 $$[\sin \,{\theta }_{t}(x)-\,\sin \,{\theta }_{i}(x)]{k}_{0}=\frac{d{\rm{\Phi }}(x)}{dx},$$

where ${\theta }_{t}$ and ${\theta }_{i}$ are transmitted angle and incident angle, respectively. ${\rm{\Phi }}(x)$ is the phase accumulation across the metasurface, *x* is transversal coordinate along the metasurface and ${k}_{0}$ represents the wavenumber in air. The transmitted angle is given by

4 $$\sin \,{\theta }_{t}(x)=\frac{1}{{k}_{0}}\frac{d{\rm{\Phi }}(x)}{dx}+\,\sin \,{\theta }_{i}(x).$$

It can be expected from Eq. (4) that the transmitted angle can be yielded by manipulating phase gradient $\xi =d{\rm{\Phi }}(x)/dx$ or incident angle ${\theta }_{i}(x)$ appropriately. In the following paragraphs, we will demonstrate acoustic metasurface capable of generating five distinct wavefront modulations: anomalous refraction, acoustic cloak based on flat focusing, acoustic self-bending beam, conversion of propagating wave to surface wave and negative refraction.

### Anomalous refraction

**Figure 3:**
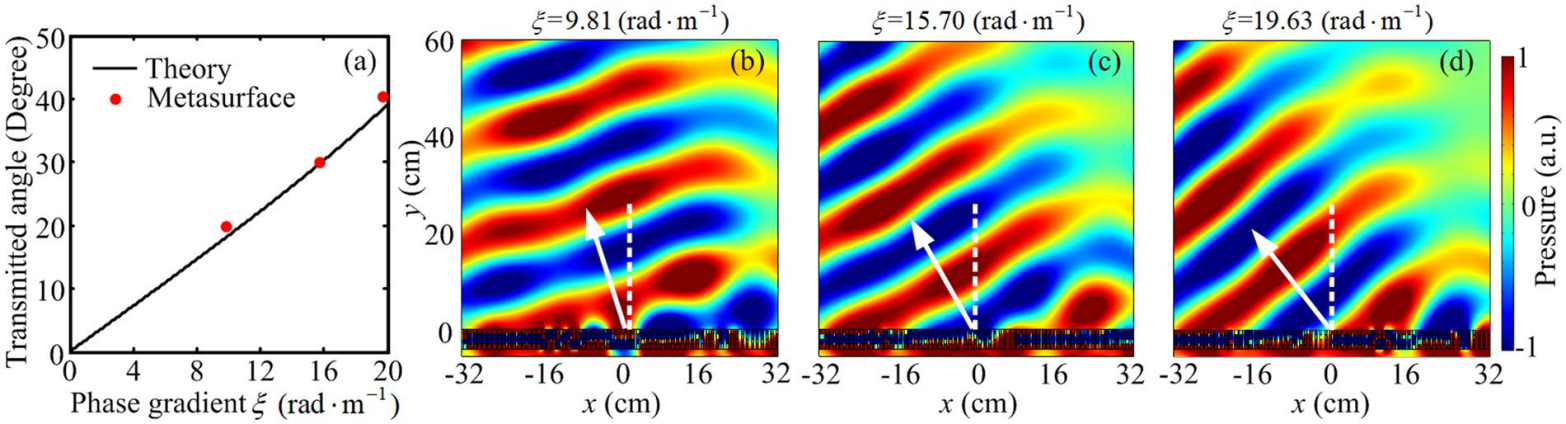
Acoustic metasurface for the anomalous refraction. (**a**) The transmitted angles as a function of phase gradient $\xi$ at normal incidence. (**b–d**) The simulated pressure field distributions of the gradient metasurfaces with phase gradients $\xi =9.81\,(\text{rad}\cdot {{\rm{m}}}^{-1})$, $\xi =15.70\,(\mathrm{rad}\cdot {{\rm{m}}}^{-1})$ and $\xi ={\rm{19.63}}\,(\mathrm{rad}\cdot {{\rm{m}}}^{-1})$, respectively.

We first demonstrate the capability of generating anomalous refraction for the designed metasurfaces. Equation (4) indicates that the transmitted angle can be tuned by properly selecting phase gradient along the metasurface. Here, we examine the case of a plane acoustic wave normally impinging onto the metasurface along the +*y* direction, three metasurfaces with different phase gradients are investigated through theory and simulation methods. Figure 3(a) plots the simulated results of the transmitted angles for the metasurfaces with phase gradients $\xi =9.81\,(\text{rad}\cdot {{\rm{m}}}^{-1})$, $\xi ={\rm{15.70}}\,(\mathrm{rad}\cdot {{\rm{m}}}^{-1})$ and $\xi =19.63\,(\mathrm{rad}\cdot {{\rm{m}}}^{-1})$, and compares them with the theoretically calculated results by the generalized Snell’s law. It is found that the simulated transmitted angles (red dots) are agree with the theoretical values (black curve). Figure 3(b–d) illustrate the simulated results of pressure field patterns for the metasurfaces with these three different phase gradient profiles at the working frequency of 1735 Hz. The theoretical transmitted angles ${\theta }_{t}$ for these cases should to be 17.94°, 29.53° and 38.05°, respectively (shown as white arrows), which are deduced from Eq. (4). As shown in Fig. 3(b–d), the refracted beams deflect from the incident directions, and the phenomenon of anomalous refraction can be clearly observed after imposing the discrete phase profile along the metasurface.

### Acoustic cloak based on flat focusing

**Figure 4:**
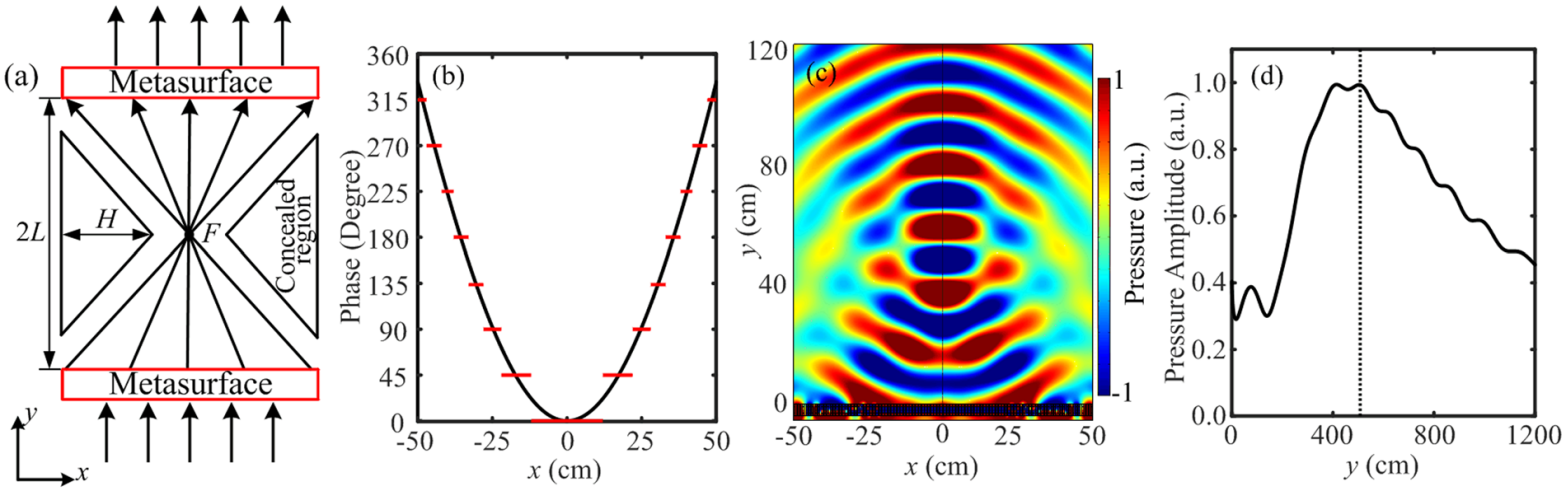
Acoustic metasurface for the flat focusing. (**a**) The concept schematic of the design of acoustic cloak based on flat focusing. (**b**) The phase distributions for the ideal metasurface (black solid curve) and gradient metasurface (red solid lines) along the *x* direction. (**c**) The simulated pressure field pattern of the gradient metasurface under normal incidence. (**d**) Normalized pressure amplitude distribution along the *y*-axis at the focal point.

The gradient metasurfaces with the property of flat focusing have been used for electromagnetic cloaks^33^. Such cloaks can easily realize cloaking for electrically large object with small thickness. Here, since our gradient acoustic metasurface has the properties of subwavelength thickness and high transmission efficiency simultaneously. Acoustic cloak can be also constructed by combining two identical flat focusing lenses. Figure 4(a) shows the concept schematic of the proposed acoustic cloak, two identical metasurfaces place symmetrically about the focus spot *F* with a focal length *L* = 59.3 cm. In order to construct an acoustic cloak, the corresponding phase profiles for these two metasurfaces can be calculated as ${\rm{\Phi }}(x)=2\pi (\sqrt{{x}^{2}+{(L)}^{2}}-L)/\lambda$. When a plane wave normally impinges on the below metasurface along the +*y* direction, the transmitted acoustic wave is focused at the center position *F*, and after that the focused acoustic wave spreads out in the form of cylindrical wave. According to reciprocity principle, when the acoustic wave passes through the upper focusing lens, the cylindrical wave is reconverted into plane wave. We note that there are two identical triangle regions (as shown in Fig. 4(a)) between the two metasurfaces, where the acoustic wave cannot reach. Thus, the object is concealed for these two regions and cloak can realize invisibility for acoustically objects. In Fig. 4(b), we present the ideal phase profile for the metasurface with a focal length *L* = 59.3 cm, as shown by the black solid curve. Using our gradient metasurface, the ideal phase profile will be discretized into eight stepwise zones at the working frequency of 1735 Hz, as shown by the red solid lines in Fig. 4(b). First, we measure the practical focal position for the single gradient metasurface by the finite element method. Figure 4(c) illustrates the simulated pressure field pattern of the gradient metasurface under normal incidence with wavelength $\lambda =19.8\,{\rm{cm}}$. It is found that the incident plane wave is efficiently transmitted and the transmitted wave is focused at a focal spot with high amplitude. To accurately measure the focal length *L*, the simulated normalized pressure amplitude distribution along the *y*-axis at the focal point with the metasurface is presented in Fig. 4(d). The focal length of the gradient metasurface is *y* = 57 cm ($\approx L$) which is similar to the theoretical result.

**Figure 5:**
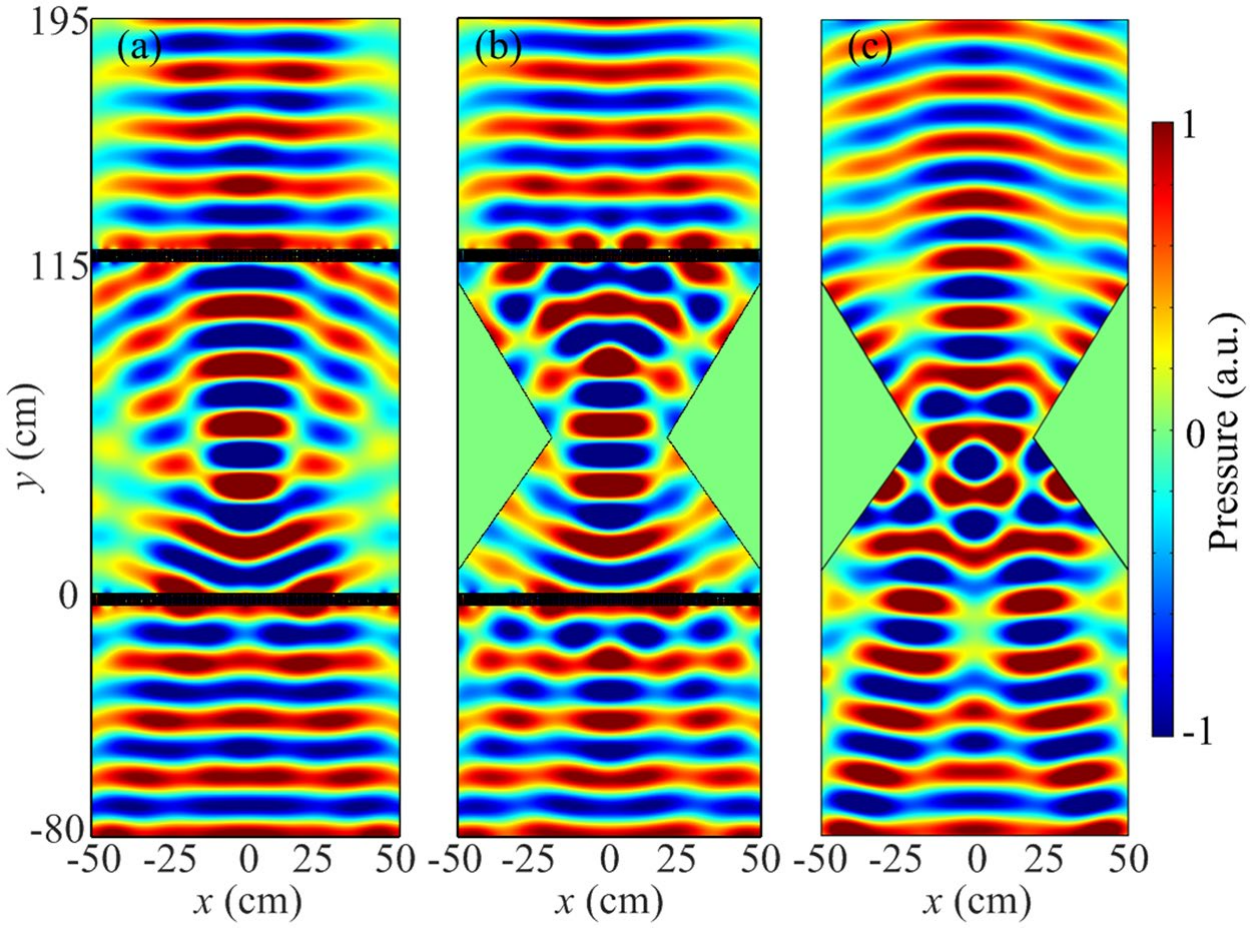
Acoustic cloak constructed by the acoustic metasurface based on flat focusing. (**a–c**) The simulated pressure field patterns of the acoustic cloak without triangle metal blocks, with triangle metal blocks and without gradient metasurfaces, respectively.

An acoustic cloak can be designed by using two identical flat focusing lenses, which successfully hides the object along the *y* direction. In order to validate the cloaking effect of this acoustic cloak, we numerically demonstrate the pressure distributions for the designed acoustic cloak with and without triangle metal blocks, the distance between two gradient metasurfaces is 115 cm. As shown in Fig. 5(a), the simulated pressure field pattern of the acoustic cloak without triangle metal blocks confirms the principle diagram in Fig. 4(a), in which we observe that the incident plane wave converts into cylindrical wave and focuses at a spot. Then the cylindrical wave radiates by the point source and reconverts into plane wave. Figure 5(b) gives the simulated pressure field pattern of the acoustic cloak with triangle metal blocks. The height of two triangle metal blocks is *H* = 31 cm. It is observed that the pressure field distribution with triangle metal blocks is well consistent with those without triangle metal blocks. This result indicates that the triangle metal blocks have almost no influence on the acoustic wave propagation between the two flat focusing lenses and the object placed in the triangle metal block becomes invisible for the acoustic wave. To verify the cloaking effect based on flat focusing, we remove the metasurfaces and retain the triangle metal blocks. The simulated pressure field pattern of the structure under normal incidence is shown in Fig. 5(c). It can be found that the incident plane wave is converted into cylindrical wave due to the geometry of the triangle metal blocks. Consequently, the acoustic cloak can be designed by the gradient metasurface based on flat focusing.

### Acoustic self-bending beam

**Figure 6:**
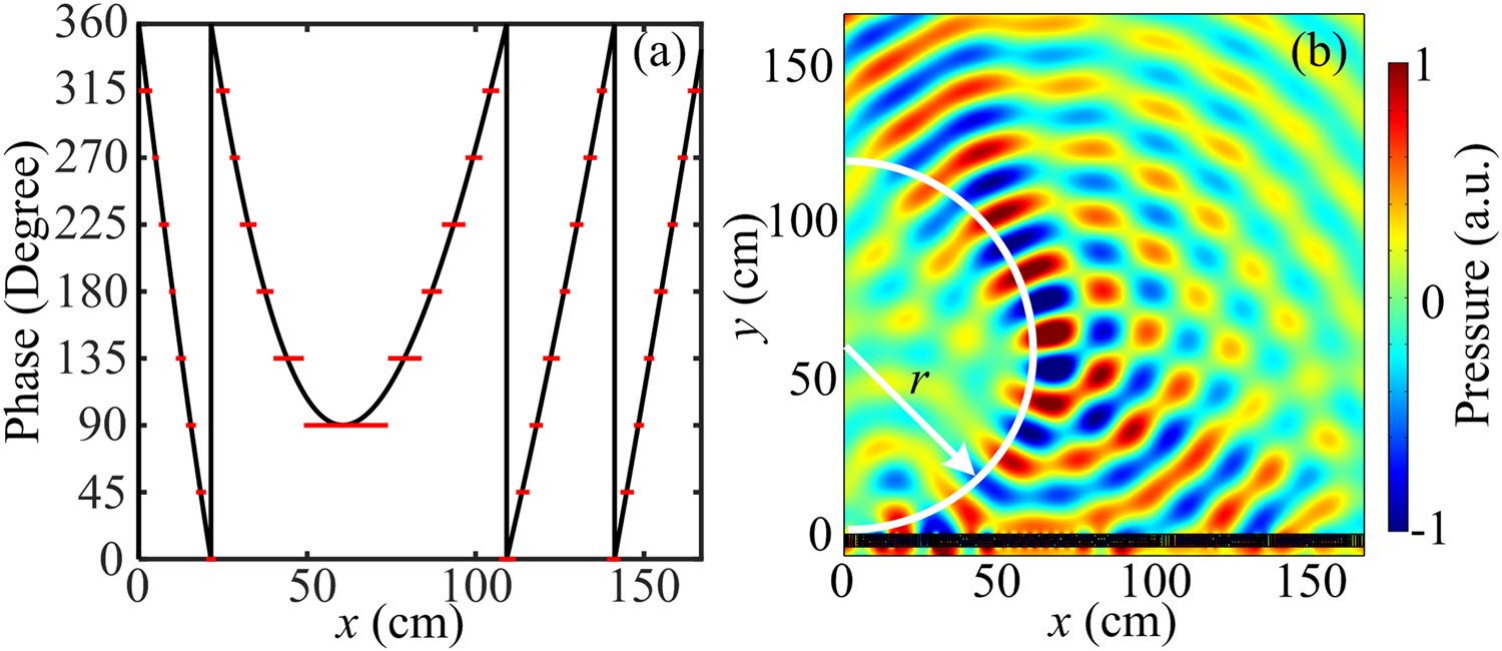
Acoustic metasurface for the generation of acoustic self-bending beam. (**a**) The phase distributions for the ideal metasurface (black solid curve) and gradient metasurface (red solid lines) along the *x* direction. (**d**) The simulated pressure field pattern of the gradient metasurface.

In the following, we will demonstrate the generation of acoustic self-bending beam. In order to generate an acoustic self-bending beam whose path is a half circle with radius *r*, the acoustic beam trajectory should satisfy $x=f(y)=\sqrt{{r}^{2}-{(y-r)}^{2}}$ with the center at (0, *r*), and the transmitted angle should satisfy $\tan \,{\theta }_{t}=f^{\prime} (y)$. Therefore, the phase profile for the ideal metasurface can be obtained as ${\rm{\Phi }}(x)=-\,{k}_{0}(|x|-2r\,\arctan (|x|/r))$. Figure 6(a) shows the phase distributions for the ideal metasurface (black solid curve) and the gradient metasurface (red solid lines) with *r* = 60.6 cm. Figure 6(b) illustrates the simulated pressure field pattern of the gradient metasurface with incident wavelength $\lambda =19.8\,{\rm{cm}}$. The distribution of the elements along the gradient metasurface is illustrated by the discrete phase shifts shown by the red solid lines in Fig. 6(a). It is observed that the transmitted acoustic wave propagates along an arc trajectory which is similar to the theoretical arc curve (drawn in white curve). Due to the periodic distribution along the *x* direction at an order smaller than the incident wavelength, good agreement is achieved between the theoretical prediction and the numerical simulation results.

### Conversion of propagating wave to surface wave and negative refraction

**Figure 7:**
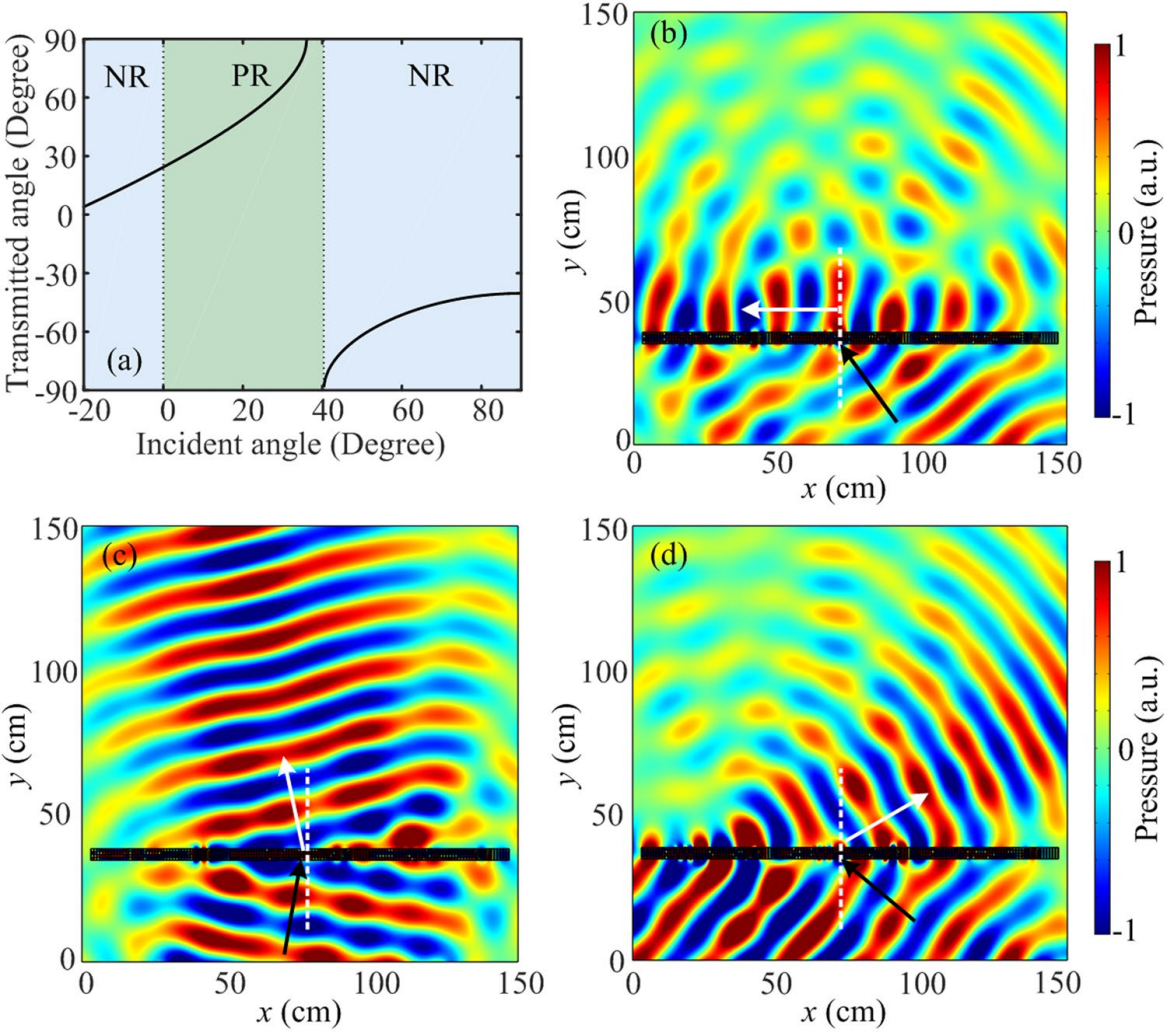
Conversion of propagating wave to surface wave and negative refraction. (**a**) The relationship between the values of transmitted angles and incident angles when the gradient metasurface with phase gradient $\xi =13.09\,({\rm{rad}}\cdot {{\rm{m}}}^{-1})$. (**b–d**) The simulated pressure field patterns for the incident angles 36°, −10° and 50°, respectively.

So far, we have presented the effect of the phase gradient on the transmissive wavefront, which does not investigate the dependence of incident angle. From Eq. (4), it is found that the transmitted angle ${\theta }_{t}$ could be manipulated by the phase gradient $\xi$ or the incident angle ${\theta }_{i}$. In the following, we will present the influence of the incident angle for the designed metasurface with a fixed phase gradient of $\xi =13.09\,({\rm{rad}}\cdot {{\rm{m}}}^{-1})$. As expected from Eq. (4), a critical incident angle ${\theta }_{{\rm{c1}}}={36}^{{\rm{o}}}$ could be obtained for the transmitted angle ${\theta }_{t}{=\mathrm{90}}^{{\rm{o}}}$. It should be noted that Eq. (4) is generally only used in the case of ${\theta }_{i}\le {\theta }_{{\rm{c1}}}$. In the region of ${\theta }_{i} > {\theta }_{{\rm{c1}}}$, such scalar diffraction theory cannot provide a description for the higher order diffraction. Under this condition, the possible supported order can be simply found by checking the incident angle with equation of $\sin \,{\theta }_{i}(x)=\,\sin \,{\theta }_{t}(x)-1/{k}_{0}(\xi +{n}_{G}G)$, where $G=2\pi /W$ is the amplitude of the reciprocal lattice vector, *W* is the periodicity of the metasurface and ${n}_{G}$ is the order of the diffraction^21^. When ${\theta }_{t}=-\,{90}^{{\rm{o}}}$, only ${n}_{G}=-\,{\rm{5}}$ can give a physically meaningful value of the critical incident angle ${\theta }_{{\rm{c}}2}={{\rm{40}}}^{{\rm{o}}}$. Generally, when the incident angle ${\theta }_{i}$ approaches a critical incident angle, the transmitted wave will propagate toward the surface and then become evanescent on the transmissive wavefront. Therefore, when the incident angle is between the first and the second critical angles (${\theta }_{{\rm{c1}}}$ and ${\theta }_{{\rm{c2}}}$), the evanescent surface mode generated is a surface wave. Figure 7(a) presents the transmitted angle as a function of incident angle range from $-{20}^{{\rm{o}}}$ to 90°. The curve exhibits an abrupt change when incident angle crosses the critical angles of ${\theta }_{{\rm{c1}}}={36}^{{\rm{o}}}$ and ${\theta }_{{\rm{c2}}}={40}^{{\rm{o}}}$. Moreover, many characteristics of the metasurface can be conveniently obtained from the curve. For the incident angle ranges $-{20}^{{\rm{o}}} < {\theta }_{i} < {0}^{{\rm{o}}}$ and ${40}^{{\rm{o}}} < {\theta }_{i} < {90}^{{\rm{o}}}$, the incident wave and transmitted wave are on the same side of the normal, indicating ranges of negative refraction (NR). The incident wave and transmitted wave on both sides of the normal are observed for the incident angle range ${0}^{{\rm{o}}} < {\theta }_{i} < {40}^{{\rm{o}}}$ (positive refraction (PR)). Therefore, the designed metasurface passes through positive and negative refraction states varying with the incident angle. Figure 7(b) gives the simulated pressure field pattern for the incident angle ${\theta }_{{\rm{c1}}}$, it is found that the surface wave is propagating near the metasurface along the -*x* direction (shown as the white arrow) after the incident wave impinging on the metasurface with angle ${\theta }_{{\rm{c1}}}$. In Fig. 7(c) and (d), we present the simulated pressure field patterns for the incident angles $-{10}^{{\rm{o}}}$ and 50° (shown as the black arrows). The refracted beams deflect to the same side of the incident beams, and the angles of the transmitted waves are around $-{12}^{{\rm{o}}}$ and 60°, which are similar to the theoretical refracted angles (shown as the white arrows).

## Discussion

In conclusion, we have theoretically designed and demonstrated a gradient metasurface with the ability of controlling transmissive wavefront. On the basis of the membrane-type hybrid structure of four decorated membrane resonators and a straight pipe, the gradient metasurface is shown to have the properties of full range of phase shift and high transmission efficiency. By employing transversal gradient phase profile along the metasurface or incident angle based on the generalized Snell’s law, the various transmissive wavefront manipulations, such as anomalous refraction, acoustic cloak based on flat focusing, acoustic self-bending beam, conversion of propagating wave to surface wave and negative refraction, have been demonstrated. The proposed gradient metasurface reveals an effective control of transmissive wavefront with high transparency, which may have potential application in low-loss acoustic devices.

## Method

Throughout the paper, the numerical simulations are conducted with the ‘Acoustic-Solid Interaction Module’ in COMSOL Multiphysics. The design of metasurface is based on the theoretical analysis. The materials applied in the numerical simulations are air, membranes and rigid walls (sound hard boundaries). A fixed constraint boundary condition is set for the both edges of the 1-D membrane. Plane wave radiation boundary condition is imposed on the incident boundaries and the periodic boundary condition is employed in the *x* direction to calculate the phase shift of the transmitted wave for different cavity widths of *w*_1_ (Fig. 2(a)) and the pressure field distributions (Fig. 2(b)). For Figs. 3–7, the plane wave radiation boundaries are imposed with an incident wave on the incidence boundaries. The remaining boundaries of the calculating area are set to the radiation boundary condition according to the wave shapes.

## Electronic supplementary material

Supplementary Information
