# Supplementary material for: Wavefront manipulation based on transmissive acoustic metasurface with membrane-type hybrid structure
Source: Sci Rep. 2018 Sep 21;8:14171. doi: 10.1038/s41598-018-32547-3 (PMC6155023; doi:10.1038/s41598-018-32547-3)
Supplement: Supplementary file 1 — Supplementary Information [file 41598_2018_32547_MOESM1_ESM.pdf]

Supplementary Information: Wavefront manipulation based on transmissive acoustic metasurface with membrane-type hybrid structure

Jun Lan[1], Xiaowei Zhang[1], Xiaozhou Liu[1,a)] , Yifeng Li[2,b)]

[1]*Key Laboratory of Modern Acoustics, Institute of Acoustics and School of Physics, Collaborative Innovation Center of Advanced Microstructures, Nanjing University, Nanjing 210093, P. R. China*

[2]*College of Computer Science and Technology, Nanjing Tech University, Nanjing 211800, P. R. China*

1. The acoustic impedance of the 1-dimentional membrane

In this paper, the 1-dimentional (1-D) membrane is modeled as a thin plate, which is characterized by its mass density ρ_{m}, Young's modulus *E*, Poisson's ratio ν, thickness *d* and width *l*. The transverse displacement of the 1-D membrane ξ satisfies the flexural wave equation[1]

$$\nabla^4\xi - k_{\mathrm{m}}^4\xi = \frac{\Delta p}{D}, \tag{1}$$

where $k_{\mathrm{m}}^2 = \omega\sqrt{\rho_{\mathrm{m}}''/D}$ and $\rho_{\mathrm{m}}'' = \rho_{\mathrm{m}} d$, $\Delta p = p_1 - p_2$ is the pressure difference across the membrane, p_1 and p_2 are the acoustic pressure on the both sides of the 1-D membrane. *D* represents the flexural rigidity, which is given by

$$D = \frac{Ed^3}{12(1-\nu^2)}. \tag{2}$$

According to factorization method, Eq. (1) can be rewritten as

$$(\nabla^2 + k_{\mathrm{m}}^2)(\nabla^2 - k_{\mathrm{m}}^2)\xi = \frac{\Delta p}{D}. \tag{3}$$

Here, the 1-D membrane is placed along the *y*-axis and clamped at its edges to a straight pipe, as shown in Fig. 1s. Assuming a uniform pressure distribution across the membrane, the following general solution for the transverse displacement $\xi(y)$ can be obtained

$$\xi(y) = -\frac{\Delta p}{k_{\mathrm{m}}^4 D} + A\cos(k_{\mathrm{m}}y) + B\sin(k_{\mathrm{m}}y) + E\mathrm{e}^{k_{\mathrm{m}}y} + F\mathrm{e}^{-k_{\mathrm{m}}y}. \tag{4}$$

The 1-order differential coefficient of $\xi(y)$ can be expressed as

$$\frac{d\xi(y)}{dx} = -Ak_{\mathrm{m}}\sin(k_{\mathrm{m}}y) + Bk_{\mathrm{m}}\cos(k_{\mathrm{m}}y) + Ek_{\mathrm{m}}\mathrm{e}^{k_{\mathrm{m}}y} - Fk_{\mathrm{m}}\mathrm{e}^{-k_{\mathrm{m}}y}, \tag{5}$$

where *A*, *B*, *E* and *F* represent the four constants. As shown in Fig. 1s, there are four boundary

conditions corresponding to the 1-D clamped membrane: $\xi(y=l)=0$, $\frac{d\xi}{dy}|_{y=l}=0$, $\xi(y=0)=0$, and $\frac{d\xi}{dy}|_{y=0}=0$. By substituting these four boundary conditions into Eq. (4), which is given by

$$\begin{cases} A+E+F=\dfrac{\Delta p}{k_{\mathrm{m}}^4 D} & (\xi(y=0)=0) \\ A\cos(k_{\mathrm{m}}l)+B\sin(k_{\mathrm{m}}l)+E\mathrm{e}^{k_{\mathrm{m}}l}+F\mathrm{e}^{-k_{\mathrm{m}}l}=\dfrac{\Delta p}{k_{\mathrm{m}}^4 D} & (\xi(y=l)=0) \\ Bk_{\mathrm{m}}+Ek_{\mathrm{m}}-Fk_{\mathrm{m}}=0 & (d\xi/dy\,(y=0)=0) \\ -Ak_{\mathrm{m}}\sin(k_{\mathrm{m}}l)+Bk_{\mathrm{m}}\cos(k_{\mathrm{m}}l)+Ek_{\mathrm{m}}\mathrm{e}^{k_{\mathrm{m}}l}-Fk_{\mathrm{m}}\mathrm{e}^{-k_{\mathrm{m}}l}=0 & (d\xi/dy\,(y=l)=0) \end{cases} \tag{6}$$

Equation (6) can be rewritten as

$$\begin{pmatrix} 1 & 0 & 1 & 1 \\ \cos(k_{\mathrm{m}}l) & \sin(k_{\mathrm{m}}l) & \mathrm{e}^{k_{\mathrm{m}}l} & \mathrm{e}^{-k_{\mathrm{m}}l} \\ 0 & k_{\mathrm{m}} & k_{\mathrm{m}} & -k_{\mathrm{m}} \\ -k_{\mathrm{m}}\sin(k_{\mathrm{m}}l) & k_{\mathrm{m}}\cos(k_{\mathrm{m}}l) & k_{\mathrm{m}}\mathrm{e}^{k_{\mathrm{m}}l} & -k_{\mathrm{m}}\mathrm{e}^{-k_{\mathrm{m}}l} \end{pmatrix} \begin{pmatrix} A \\ B \\ E \\ F \end{pmatrix} = \begin{pmatrix} 1 \\ 1 \\ 0 \\ 0 \end{pmatrix} \frac{\Delta p}{k_{\mathrm{m}}^4 D}. \tag{7}$$

Here, since the coefficient $\Delta p/\left(k_{\mathrm{m}}^4 D\right)$ can be easily eliminated with normalization, we will assume *A*, *B*, *E*, *F* as the non-dimensional coefficient in the following discussion. Then from Eq. (7), we can obtain

$$\begin{cases} A=1-E-F \\ B=F-E \end{cases}. \tag{8}$$

By substituting Eq. (8) into Eq. (7), which gives

$$\begin{cases} (\mathrm{e}^{k_{\mathrm{m}}l}-\cos(k_{\mathrm{m}}l)-\sin(k_{\mathrm{m}}l))E+(\mathrm{e}^{-k_{\mathrm{m}}l}-\cos(k_{\mathrm{m}}l)+\sin(k_{\mathrm{m}}l))F=1-\cos(k_{\mathrm{m}}l) \\ (\mathrm{e}^{k_{\mathrm{m}}l}-\cos(k_{\mathrm{m}}l)+\sin(k_{\mathrm{m}}l))E+(-\mathrm{e}^{-k_{\mathrm{m}}l}+\cos(k_{\mathrm{m}}l)+\sin(k_{\mathrm{m}}l))F=\sin(k_{\mathrm{m}}l) \end{cases}. \tag{9}$$

Equation (9) can be rewritten as

$$\begin{pmatrix} \mathrm{e}^{k_{\mathrm{m}}l}-\cos(k_{\mathrm{m}}l)-\sin(k_{\mathrm{m}}l) & \mathrm{e}^{-k_{\mathrm{m}}l}-\cos(k_{\mathrm{m}}l)+\sin(k_{\mathrm{m}}l) \\ \mathrm{e}^{k_{\mathrm{m}}l}-\cos(k_{\mathrm{m}}l)+\sin(k_{\mathrm{m}}l) & -\mathrm{e}^{-k_{\mathrm{m}}l}+\cos(k_{\mathrm{m}}l)+\sin(k_{\mathrm{m}}l) \end{pmatrix} \begin{pmatrix} E \\ F \end{pmatrix} = \begin{pmatrix} 1-\cos(k_{\mathrm{m}}l) \\ \sin(k_{\mathrm{m}}l) \end{pmatrix}. \tag{10}$$

Therefore, the four constants of Eq. (4) can be expressed as

$$\begin{cases} E = \dfrac{b_1 \times a_4 - b_2 \times a_2}{a_1 \times a_4 - a_2 \times a_3} \\ F = \dfrac{b_2 \times a_1 - b_1 \times a_3}{a_1 \times a_4 - a_2 \times a_3} \\ A = 1 - E - F \\ B = F - E \end{cases}, \tag{11}$$

where $a_1 = e^{k_{\mathrm{m}}l} - \cos(k_{\mathrm{m}}l) - \sin(k_{\mathrm{m}}l)$, $a_2 = \mathrm{e}^{-k_{\mathrm{m}}l} - \cos(k_{\mathrm{m}}l) + \sin(k_{\mathrm{m}}l)$, $a_3 = \mathrm{e}^{k_{\mathrm{m}}l} - \cos(k_{\mathrm{m}}l) + \sin(k_{\mathrm{m}}l)$, $a_4 = -\mathrm{e}^{-k_{\mathrm{m}}l} + \cos(k_{\mathrm{m}}l) + \sin(k_{\mathrm{m}}l)$, $b_1 = 1 - \cos(k_{\mathrm{m}}l)$ and $b_2 = \sin(k_{\mathrm{m}}l)$. Generally, the acoustic impedance of the 2-dimentional circular membrane can be defined as $Z_{\mathrm{am}} = \iint_S \Delta p dS \big/ \left(j\omega\bar{\xi}S^2 \right)$, In our 1-D membrane, the cross-sectional area of the membrane *S* is the width of the 1-D membrane *l*. Therefore, the acoustic impedance of the 1-D membrane is given by

$$Z_{\mathrm{am}} = \frac{\int_l \Delta p(y) dl}{j\omega\bar{\xi}l^2}, \tag{12}$$

where $\bar{\xi} = (1/l)\int_l \xi(y)dl$ is the average transverse displacement over the membrane surface. Here, assuming a uniform pressure distribution over the membrane, by substituting Eq. (4) into Eq. (12), the acoustic impedance of the 1-D membrane can be given by

$$\begin{aligned} Z_{\mathrm{am}} = \frac{\int_l \Delta p(y) dl}{j\omega\bar{\xi}l^2} &= \frac{\Delta pl}{j\omega \dfrac{\Delta p}{k_{\mathrm{m}}^4 D} \dfrac{1}{l} \int_0^l -1 + A\cos(k_{\mathrm{m}}y) + B\sin(k_{\mathrm{m}}y) + E\mathrm{e}^{k_{\mathrm{m}}y} + F\mathrm{e}^{-k_{\mathrm{m}}y} dl \times l^2} \\ &= \frac{k_{\mathrm{m}}^4 D}{j\omega \left[-y + \dfrac{A}{k_{\mathrm{m}}}\sin(k_{\mathrm{m}}y) - \dfrac{B}{k_{\mathrm{m}}}\cos(k_{\mathrm{m}}y) + \dfrac{E}{k_{\mathrm{m}}}\mathrm{e}^{k_{\mathrm{m}}y} - \dfrac{F}{k_{\mathrm{m}}}\mathrm{e}^{-k_{\mathrm{m}}y} \right]\Bigg|_0^l} \\ &= \frac{k_{\mathrm{m}}^4 D}{j\omega \left[-l + \dfrac{A}{k_{\mathrm{m}}}\sin(k_{\mathrm{m}}l) - \dfrac{B}{k_{\mathrm{m}}}\cos(k_{\mathrm{m}}l) + \dfrac{B}{k_{\mathrm{m}}} + \dfrac{E}{k_{\mathrm{m}}}\mathrm{e}^{k_{\mathrm{m}}l} - \dfrac{E}{k_{\mathrm{m}}} - \dfrac{F}{k_{\mathrm{m}}}\mathrm{e}^{-k_{\mathrm{m}}l} + \dfrac{F}{k_{\mathrm{m}}} \right]} \end{aligned} \tag{13}$$

where the expressions of *A*, *B*, *E* and *F* have been presented in Eq. (11). Therefore, the effective acoustic impedance of the 1-D membrane can be derived from Eq. (13), and the membrane provides an effective acoustic reactance to shift the phase of the incident acoustic wave over the whole 2π range and realize the highly efficient transmission.

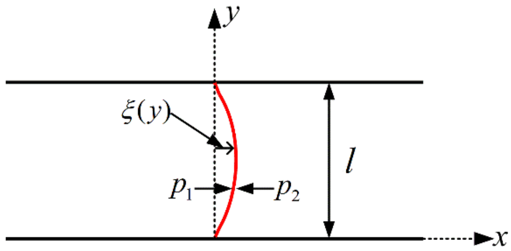

Figure 1s. 1-dimentional membrane clamped to a straight pipe. Red solid line refers to the 1-dimentional membrane.

Supplementary References

1. Bongard, F., Lissek, H. & Mosig, J. R. Acoustic transmission line metamaterial with negative/zero/positive refractive index. *Phys. Rev. B* **82**, 94306 (2010).
